# Corticospinal excitability for hand muscles during motor imagery of foot changes with imagined force level

**DOI:** 10.1371/journal.pone.0185547

**Published:** 2017-09-28

**Authors:** Kouki Kato, Kazuyuki Kanosue

**Affiliations:** Faculty of Sport Sciences, Waseda University, Tokorozawa, Japan; Nanjing Normal University, CHINA

## Abstract

The object of this study was to clarify whether corticospinal excitability controlling hand muscles changes concurrently with increases in the imagined contraction level of foot dorsiflexion. Twelve participants performed actual and imagined dorsiflexion of their right foot at three different EMG levels (10, 40 or 80% of the maximum voluntary contraction). During isometric actual- or imagined- dorsiflexion, transcranial magnetic stimulation (TMS) was delivered to the right hand area of the left primary motor cortex. Motor evoked potentials (MEPs) were recorded from the right extensor carpi radialis (ECR) and flexor carpi radialis (FCR). During actual contraction, MEP amplitudes of ECR and FCR increased with an increased EMG level of dorsiflexion. Similarly, during imagery contraction, MEP amplitudes of ECR and FCR increased with the intensity of imagery contraction. Furthermore, a correlation between MEP amplitude during actual contraction and imagery contraction was observed for both ECR and FCR. Motor imagery of foot contraction induced an enhancement of corticospinal excitability for hand muscles that was dependent on the imagined contraction levels, just as what was observed when there was an actual contraction.

## Introduction

Motor imagery is the mental representation of an action without any overt movement or muscle activation [1]. Practice involving motor imagery is widely used in both sports and rehabilitation, and many studies have demonstrated beneficial effects for improving motor skills as well as rehabilitating neurological impairments [2,3,4,5,6,7,8,9]. During motor imagery, brain regions such as the primary motor cortex (M1), premotor cortex, supplemental motor area, cerebellum, and basal ganglia are activated in a way similar to that which occurs during actual task execution [10,11,12,13,14]. Furthermore, many studies have demonstrated that motor imagery of muscle contraction induces an enhancement of corticospinal excitability, as assessed by a comparison with motor evoked potentials (MEPs) evoked by transcranial magnetic stimulation (TMS) for the target muscle itself [11,15,16,17,18]. A recent study has shown that, during motor imagery, the corticospinal excitability associated with a particular muscle changed depending upon the force level of the imagined contraction [14].

Many approaches have confirmed a relationship between brain activity and force level of the actual muscle contraction [19,20,21,22]. Studies utilizing single-cell recording in monkeys indicate that firing rates of motor-related cortical neurons, such as those in the contralateral M1 and the premotor cortex, increase with an increase in the force level of contraction [19,20,21]. Neuroimaging studies utilizing functional magnetic resonance imaging (fMRI) in humans have also demonstrated that the level of activation of motor-related cortical regions is dependent upon the force level of muscle contraction [22,23]. Corticospinal excitability has also been shown to increase concurrently with changes in magnitude of the force level [24].

In the motions involved with daily life as well as those used in sports, simultaneous use of multiple limbs (e.g. both hands or a hand and a foot) is often required. It is well known that movement of one limb influences that of the others. This remote effect has been well studied [25,26,27]. Investigations using TMS demonstrate that simple muscle contraction enhances the corticospinal excitability of brain regions which control muscles in remote segments. This provides a neural basis for the remote effect [28,29,30,31,32,33,34,35]. The remote effect on coricospinal excitability is augmented by an increase in the contraction level [36]. Interestingly, “motor imagery” of muscle contraction also induces an increase in the corticospinal excitability of remote muscles [37]. Then, it could be hypothesized that corticospinal excitability is influenced by changes in the imagined force level of remote muscles. The aim of the present study was to test this hypothesis. We utilized TMS to elicit MEPs of the right hand muscles during imagery of contraction of the right foot muscle at different contraction intensity levels.

## Method

### Participants

Twelve right-handed, healthy volunteers (nine men and three women; aged 21–26 years) without known neurological or psychiatric disease participated in the experiment. All participants gave written, informed consent. The experimental procedure was approved by the Human Research Ethics Committee of Waseda University and performed according to the Declaration of Helsinki.

### Recording

Surface EMGs were recorded from the right extensor carpi radialis (ECR), flexor carpi radialis (FCR) and tibialis anterior (TA) via disposable Ag-AgCl electrodes (1 cm diameter) which were placed over the belly of the muscle. Before the electrodes were attached, the involved area of skin was shaved and treated with alcohol to reduce inter-electrode impedance. Inter-electrode impedance and EMG signals for the three muscles were checked after placing the electrodes. The EMG signal was amplified (MEB-2216, Nihonkoden, Japan) and bandpass filtered between 5 and 1500 Hz. All signals were converted into digital data via an A/D converter system which sampled at a rate of 4000 Hz.

### Procedure

**Fig 1:**
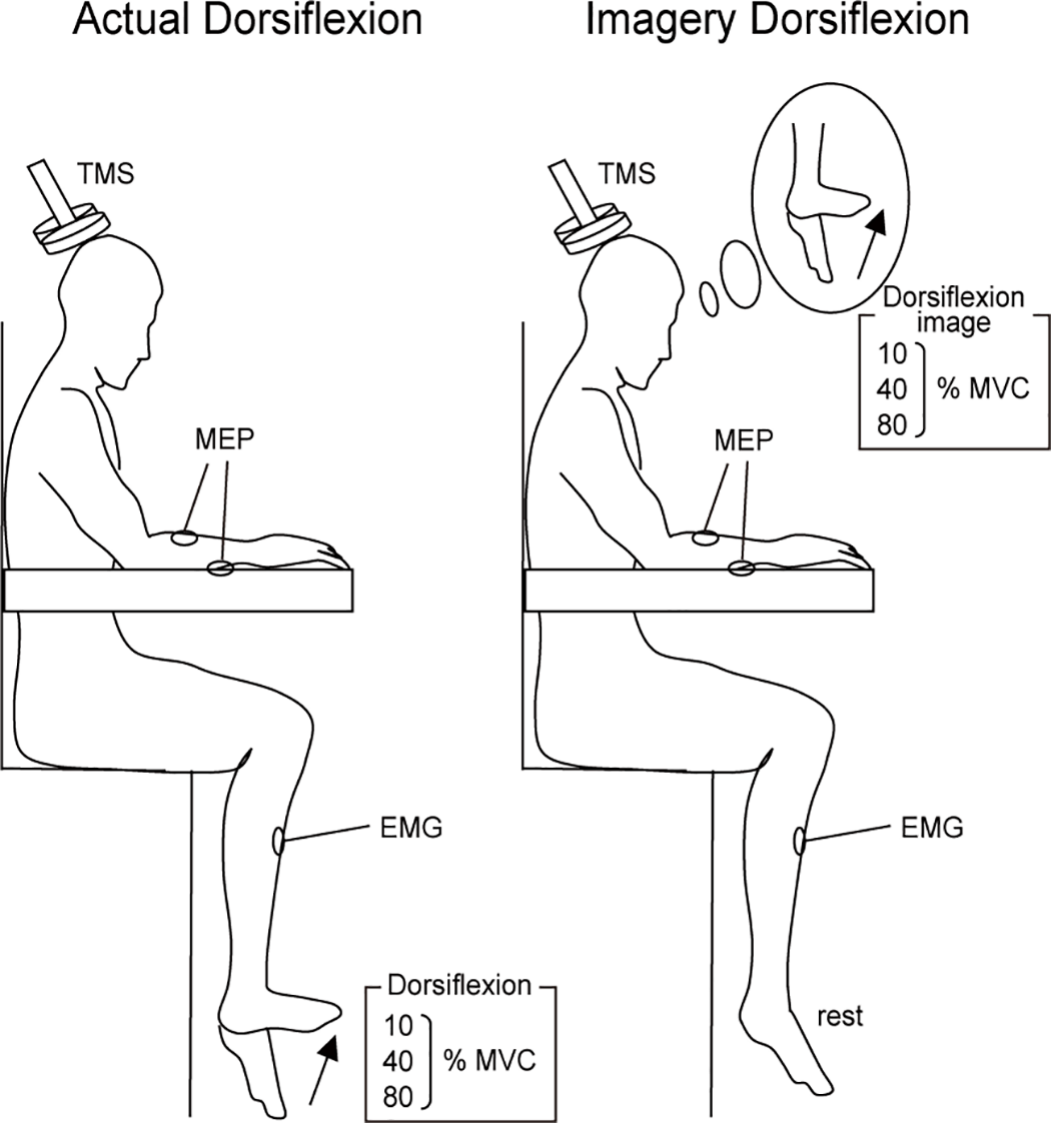
Illustration of the experimental setup. Participants performed actual and imagined dorsiflexion of their right foot at three different EMG levels (10, 40 or 80% of the maximum voluntary contraction).

The participants sat in a comfortable chair with their right forearm supported on an armrest in a horizontal position. The hand remained in a pronated position throughout the experiment. During the recordings, the participants were instructed not to activate muscles in their hands or in their left foot. The experimenter confirmed that the right foot of the participants did not touch the ground during task execution. At the beginning of the experiment, the participants were asked to perform a maximal voluntary contraction (MVC) for right foot dorsiflexion three times. Each time, they were told, and verbally encouraged during the contraction, to develop a force as hard as possible for 3s. Next, a visual line indicating the target EMG level (10, 40 or 80% of the MVC) that the participant would be asked to exert was displayed on a PC monitor, which was positioned about 1 m from the participant. The subjects practiced isometric dorsiflexion by matching the rectified and smoothed target EMG level for each MVC (10, 40 or 80%). This task was termed “actual dorsiflexion” (Fig 1 left). Then, the participants practiced imagining that they were maintaining dorsiflection in their right foot at the same intensities that were performed in the actual dorsiflexions (10, 40 and 80% MVC). This task was termed “imagery dorsiflexion” (Fig 1 right). For the motor imagery, participants were asked to use a first person perspective [10], and we confirmed that there was no EMG activity. In order to evaluate the subjects’ motor imagery ability, each participant took the Vividness of Motor Imagery Questionnaire (VMIQ) [38]. With the VMIQ, a participant rates the vividness of motor imagery on a 5-point Likert scale (1 = vivid imagery, 5 = no imagery at all). We administered this questionnaire at the end of the experiment. If the experimenter noticed unexpected EMG activity in the TA during the imagery dorsiflexion task, the practice session was extended until such EMG activity disappeared. Before the experiment, the participants performed an exertion at each EMG level (pre) without viewing the PC monitor. During the actual experiment, the participants also performed each force level exertion without viewing the PC monitor. During each break period, the participants confirmed that they were performing the exertion at the correct EMG level by viewing the PC monitor. After the trials were completed, the EMGs at the three target levels and the MVC were again recorded again (post expeirment recordings) without viewing the PC monitor as a verification test. TMS was delivered during actual isometric dorsiflexion or imagery isometric dorsiflexion. The maintenance period for actual and imagery dorsiflexion before the stimulation was varied randomly from 2 to 5 sec. In addition to being delivered during the “actual dorsiflexion” and “imagery dorsiflexion” tasks, TMS was delivered 15 times when there was no motor action or imagery as a resting control task. These three tasks were randomly mixed. There were 105 trials overall. To avoid fatigue, the inter-trial interval was always more than 20 sec. The participants took a break after every 20 tasks. The entire experiment lasted approximately 2 h.

### TMS

TMS was applied to the left M1 using a magnetic stimulator (Magstim 200, Magstim Ltd, UK) connected to a figure-eight coil (95 mm diameter) [39,40,41]. The participants wore a tight fitting swimming cap on which the position for stimulation was marked by a color marker. The coil was held by hand, and its position with respect to the marks carefully monitored. The TMS pulse was delivered to the M1 site at the best location for eliciting MEPs in both the right ECR and FCR (hot-spot) with a maximum intensity of 1.3 T. The resting motor threshold (rMT) was defined as the minimum stimulus intensity that produced an MEP amplitude with a magnitude greater than 50 μV for both the ECR and FCR for at least five out of ten stimulation trials. Stimulus intensity was set at 120% of the resting motor threshold during the experiment. The mean TMS intensity (± standard deviation, SD) was 68 ± 14% of the maximum output of the stimulator.

### Data analysis

To evaluate corticospinal excitability, peak-to-peak MEP amplitudes of the ECR and FCR were recorded, and then standardized utilizing the average value of the resting task. Background EMGs of the ECR, FCR and TA were calculated as the RMS values of the EMGs for a 50 ms window just prior to the TMS. Trials with a background EMG activity of ECR and FCR that were greater than 25 μV were considered error trials and were eliminated from the analysis. During imagery dorsiflexion (DF), if any trial involved definite activity in the TA that was greater than 25 μV, data from that trial was also excluded from the analysis. The mean percentage rate of data rejection was 1.8 ± 0.9% for the background EMG of the ECR and FCR, and 2.9 ± 1.2% for TA activity during imagery DF.

For comparison of group data, a two-factor analysis of variance (ANOVA) with repeated measures was performed for the tasks (actual DF and imagery DF) and for EMG intensity (rest, 10%, 40% and 80% DF). The significances reported for the F-values are those obtained after the Greenhouse-Geisser correction (when appropriate); then a correction coefficient ε is given (only when the degree of freedom was adjusted). For post hoc comparisons, multiple pair-wise tests with Bonferroni corrections were performed. Each dorsiflexion force of the reproduction test was normalized with reference to the mean MVC value of each participant. The MVCs and the reproduction test were evaluated with a one-sample *t* test. To investigate the relationship between MEP amplitude for the imagery DF and for the actual DF (within participants), a Pearson’s correlation coefficient between changes in MEP amplitude of the ECR for actual DF and for imagery DF was calculated for the three different EMG intensities (10, 40 and 80%). The linear regressions on the MEP amplitudes of ECR and FCR during the actual and imagery DFs were analyzed in the range of 0 to 200% of rest values to determine whether their slopes were equal to 1. The residuals of all the dots from the average were compared using a partial correlation analysis. The coefficient of variation was compared to the average of the linear regression. Subsequently the results (residual) of the actual DF and imagery DF tasks were analyzed. Significance was set at p < 0.05. The data are expressed as mean ± SD.

## Results

**Fig 2:**
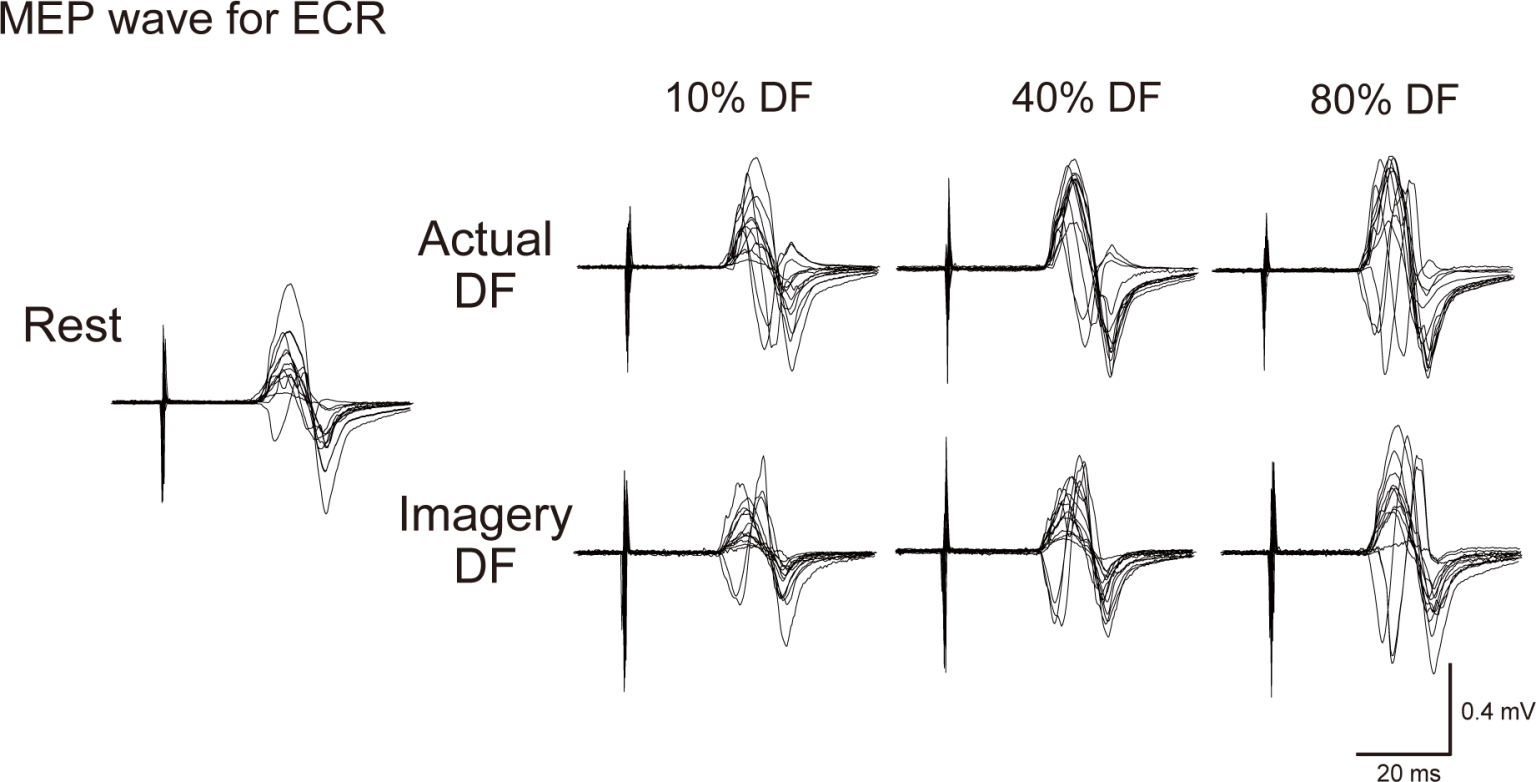
MEP waveform. Raw motor-evoked potential (MEP) waveform of the ECR elicited by single-pulse TMS in a participant at three different intensities (10, 40 and 80%) in the actual and imagery dorsiflexion (DF) task and the resting condition. Fifteen wave forms at each force level are superimposed.

**Fig 3:**
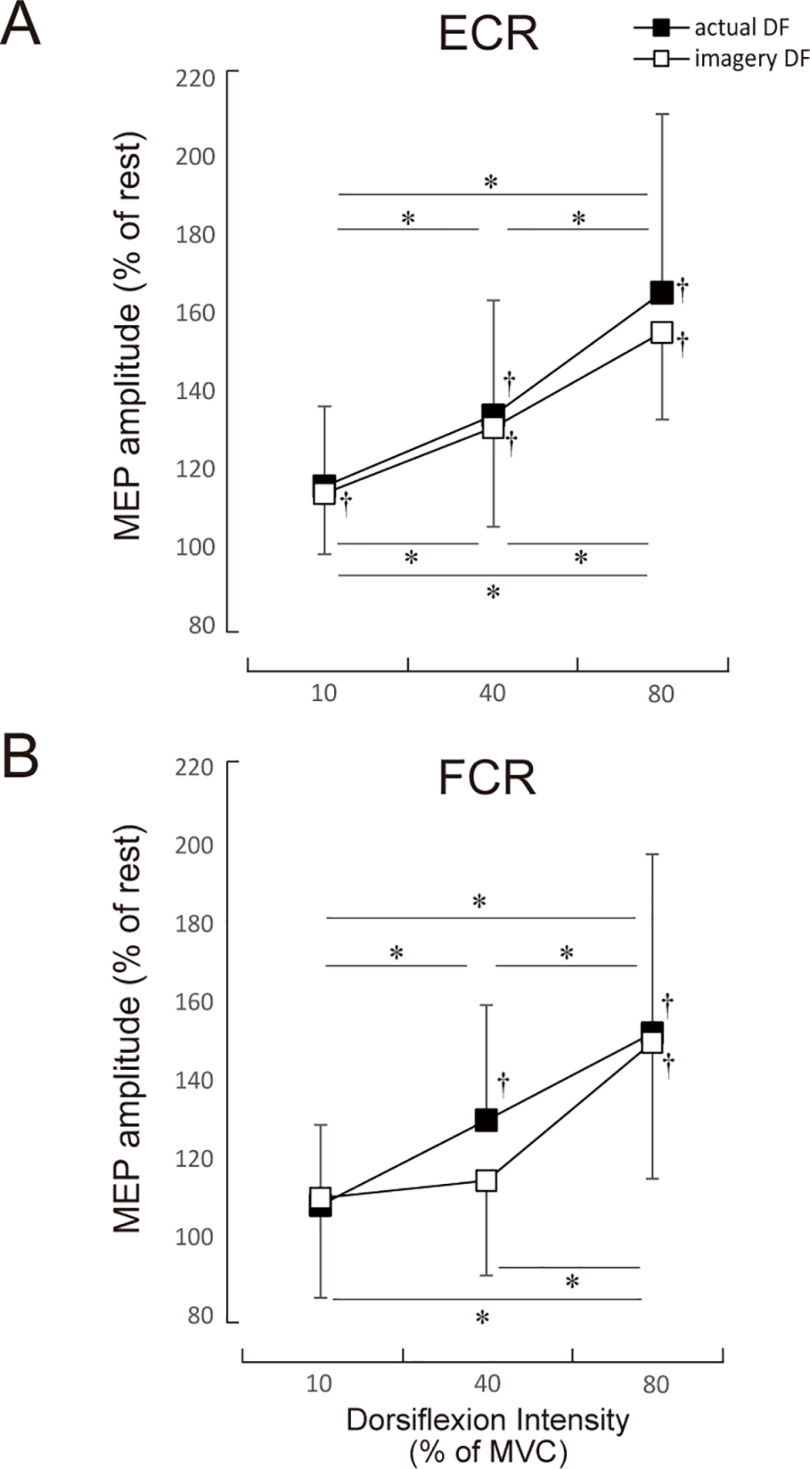
MEP amplitude of ECR and FCR. The mean MEP amplitudes and standard deviation of the ECR and the FCR during actual and imagery DF. * denotes p<0.05 levels of significance for comparison between the three intensities. † denotes p<0.01 levels of significance in a comparison with the resting condition (i.e. 100). Values are mean ± SD.

The mean scores of VMIQ for each intensity of imagery contraction were 10%; 1.8 ± 0.7, 40%; 1.9 ± 0.8, and 80%; 2.2 ± 0.7. Typical examples of MEP waves for ECR at three different EMG levels (10, 40, 80% MVC) of foot dorsiflexion during the actual and imagery dorsiflexion tasks, as well as in the resting condition, are shown in Fig 2. For both ECR and FCR, MEP amplitude was markedly increased during both actual and imagery dorsiflexion. The extent of the increase was graded for the dorsiflexion EMG levels (actual 10%: 115.7 ± 20.5% of resting condition, actual 40%: 133.9 ± 29.4, actual 80%: 165.3 ± 45.7 for ECR) (imagery 10%: 113.9 ± 15.5, imagery 40%: 130.8 ± 25.5, imagery 80%: 155.1 ± 22.3 for FCR). The grand means (± SD) for all subjects are presented in Fig 3.

For the ECR, a two-factor repeated measures ANOVA demonstrated a significant main effect for intensity (F_3, 77_ = 44.16, p < 0.001). The two-way ANOVA did not demonstrate a significant main effect for task (F_1, 77_ = 0.93, p = 0.338) or interaction of the two factors (F_3, 77_ = 0.582, p = 0.628, e = 4.595). For the actual dorsiflexion, post hoc comparisons showed no significant difference between 10% MVC and the resting condition for actual dorsiflexion (p = 0.351). For imagery dorsiflexion, however, the MEP amplitude of 10% MVC was significantly higher than that of the resting condition (p < 0.01). The MEP amplitude of 40% MVC was significantly higher than that of the resting condition and the 10% MVC was significantly higher for both the actual and imagery dorsiflexion (p < 0.01, respectively). Likewise, the MEP amplitude of 80% was significantly higher than that of the resting condition, as were the 10% and 40% MVC for both actual and imagery dorsiflexion (p < 0.01 for both).

For FCR, a two-factor repeated measures ANOVA demonstrated a significant main effect for intensity (F_3, 77_ = 21.10, p < 0.001). The two-way ANOVA did not demonstrate a significant main effect for task (F_1, 77_ = 0.69, p = 0.409) or for the interaction of the two main factors (F_3, 77_ = 0.656, p = 0.582, e = 4.332). Post hoc comparisons showed no significant differences between the 10% MVC and the resting condition for either actual or imagery dorsiflexion (p = 0.876, p = 0.530, respectively). The MEP amplitude of 40% MVC for actual dorsiflexion was significantly higher than that of the resting condition and 10% MVC (p < 0.05). However, no significant differences were observed between 40% MVC and the resting condition or 40% MVC and the 10% MVC for imagery dorsiflexion (p = 0.221, p = 0.931). For actual and imagery dorsiflexion MEP amplitudes for the 10%, 40%, and 80% MVC were significantly higher than those of the resting condition (p < 0.01 for all).

**Fig 4:**
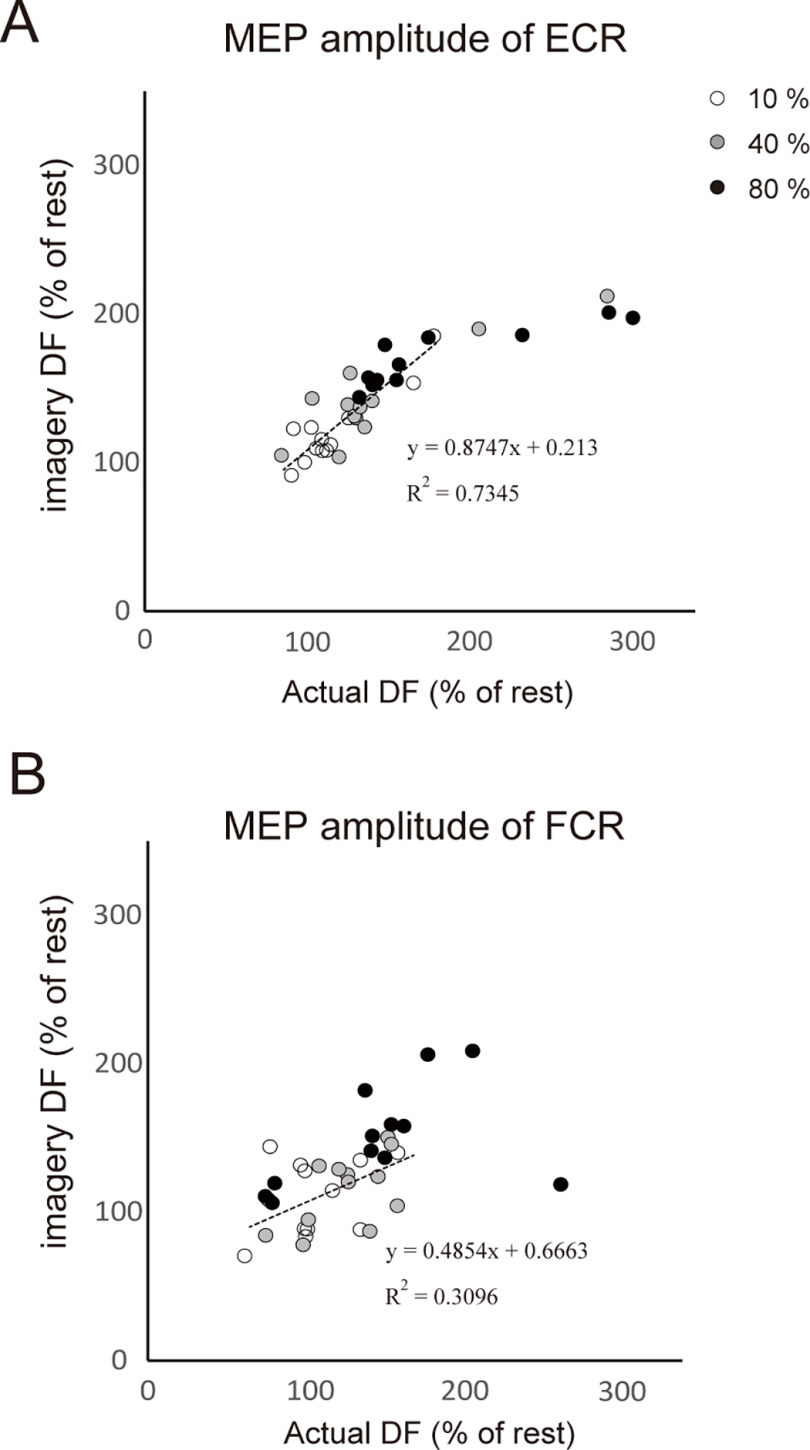
Correlation of MEP amplitude. Relationship between MEP amplitude of ECR and FCR during the imagery of DF and of the actual DF. Each circle denotes a 10% (white circle), 40% (grey circle) and 80% (black circle) of MEP for all subjects. The dotted line indicates the linear regressions in the range of 0 to 200%.

Fig 4 illustrates the relationship between the MEP amplitudes of ECR (Fig 4A) and FCR (Fig 4B), both for the actual DF and the imagery DF. A significant correlation was obtained between MEP amplitudes for the actual DF and the imagery DF of ECR (r = 0.86, p < 0.01; Fig 4A). A significant correlation was also obtained between MEP amplitudes of the actual DF and imagery DF for FCR (relaxation; r = 0.54, p < 0.05; Fig 4B). Furthermore, we also evaluated whether there was a difference between MEP amplitudes during actual DF and imagery DF over the range of 0 to 200% for resting values. For ECR, there was a close correlation between MEP amplitude during actual DF and imagery DF (Slope: a = 0.8747, R^2^ = 0.7345). This slope was not significantly different from the slope of y = x (which equals 1). For FCR, there was also a close correlation between MEP amplitude during actual DF and imagery DF (Slope: a = 0.4854, R^2^ = 0.3096). For this slope as well, there was no significant difference from the slope of y = x (which equals 1).

Participants were able to reproduce each force level (10% MVC condition; pre:12.35 ± 3.3, post: 13.19 ± 3.4%; 40% MVC condition; pre:44.52 ± 7.0, post: 42.13 ± 7.8%; 80% MVC condition; pre:75.69 ± 12.0, post: 73.53 ± 13.6%; 100% MVC condition; pre:100 ± 0, post: 93.25 ± 7.5%). The MVC in the validation test did not differ from that obtained in the pre-experiment.

## Discussion

The objective of the present study was to investigate how the level of imagined contraction in one limb influences the corticospinal excitability for the remote limb muscles. Corticospinal excitability of the hand muscles markedly increased during both actual and imagery dorsiflexion of the ipsilateral foot, and the extent of the increase was proportional to the intensity of contraction, either actual or imagined. Furthermore, correlations between MEP amplitudes during actual DF and imagery DF were observed for both the ECR and FCR.

These results suggest that the cortical mechanisms involved in the remote effect of motor imagery are similar to those involved in motor execution. The findings related to the remote effect of actual contraction correspond well with those of previous studies [36,37,42]. That is, coricospinal excitability for the upper limb muscles increases in association with increasing force levels produced by the lower limb. Mizuguchi et al [18] previously demonstrated that during motor imagery of the contraction of a hand muscle, corticospinal excitability of the muscle itself changed in a manner dependent on the force level of the imagined contraction. Studies utilizing fMRI showed that the brain regions involved with motor imagery include the supplemental motor area (SMA), pre-SMA, premotor area (PM), primary motor cortex (PMC), posterior parietal cortex (PPC), basal ganglia, and cerebellum. In addition to activation during motor imagery, these areas are also active during actual contractions [10,11,12,13,14,43]. Therefore, at a minimum, these regions are likely to be involved with the neural processing which underlies the remote effect that occurs during motor imagery. Tazoe et al. [42] noted that during an actual contraction, “the origin of the remote effect may be a higher motor centre located upstream of M1.” Since there are no anatomical connections between the hand and foot areas in M1, Brown et al. [44] and Huntley et al. [45] suggest that the supra M1 regions are involved in the remote effect. Furthermore, while M1 and SMA are arranged somatotopically, the pre-SMA is not [46,47,48]. Thus, it is possible that neural signals for the contraction of a target muscle from the pre-SMA to the SMA region might spread to the SMA that controls the remote site, although the specific neural pathways and mechanism remain unclear.

As shown by the correlation between MEP amplitudes of actual DF and imagery DF, there is a similarity, even at the individual level, between the remote effects elicited by actual and imagery contractions. Participants who showed a higher MEP amplitude of the ECR during the actual DF also showed a higher MEP amplitude during imagery DF in the MEP amplitude range less than 200% (Fig 4). Interestingly, the slope of the linear regression was not significantly different from 1. The MEP amplitude during imagery DF was saturated in the range above 200%. Likewise, a correlation with a slope close to 1 was observed for the FCR, although the correlation coefficient was smaller than that for the ECR. These results strongly suggest that a similar neural pathway is activated for the remote effect during both motor imagery and actual contraction. This is in agreement with previous studies pertaining to the similarity of brain regions involved with motor imagery and actual contraction. On the other hand, MEP amplitude of the ECR plateaued during imagery DF, while the MEP amplitude during an actual contraction did not show any apparent saturation. Thus, saturation of the MEP amplitude during imagery DF likely occurs before the signals from the imagery process are fed into the common process of the remote effect.

Remote effects that occur during an actual contraction occur both from the upper limb to the lower limb and from the lower to the upper limb [30,36,49]. Since coincidental enhancements of the H-reflex and MEPs by actual contraction of a remote site has been observed in both extensor and flexor muscles, the remote effect is thought to be non-specific [28,50]. In the present study, corticospinal excitabilities increased for both extensor and flexor muscles during an actual contraction, which corresponds well with the results of previous studies. However, we observed no significant differences between MEP amplitudes during imagery dorsiflexion of 10% and the resting condition for the FCR (Fig 3). Nor did we find any significant differences between MEP amplitudes during the imagery dorsiflexions of 10% and 40% MVC. Thus, relatively weak dorsiflexion did not alter the MEP amplitude of hand flexors. For the ECR, on the other hand, MEP amplitudes in the pronated hand position were clearly modulated by both actual and imagined contraction levels of dorsiflexion. During cyclic plantarflexion-dorsiflexion movement of the foot, MEP amplitude for the resting ECR in the pronated hand position is higher during dorsiflexion than during plantar flexion, while MEP amplitudes for the FCR is higher during plantarflexion than during dorsiflexion [51,52]. This suggests that neural activity that controls the hand extensors and flexors is affected by activity in the foot dorsiflexors and plantarflexors in a muscle specific manner. Since participants performed or imagined tonic dorsiflexion and not cyclic movements in the present study, connectivity of the remote effect might be different between muscles. Furthermore, changes in corticospinal excitability of the forehand under different positions (prone or supine) during cyclic movement of the foot has been shown to be direction-dependent and not muscle-dependent [53]. This suggests that the remote effect between the ipsilateral foot and hand might differ depending on hand position (prone or supine). We need further study to elucidate this.

In conclusion, we have provided evidence that there is an increase in corticospinal excitability of the system that provides input for the control of resting hand muscles. This increase is proportional to the imagined contraction level of the foot.
